# Role of Bean Yellow Mosaic Virus P1 and HC-Pro in Enhancing Gene Expression and Suppressing RNA Silencing in *Nicotiana benthamiana*

**DOI:** 10.3390/life15030472

**Published:** 2025-03-15

**Authors:** Sunmee Choi, Suk Hyun Kwon, Gi Seok Kwon, Ho Seong Choi, Hyo Hyun Seo, Young Soon Kim, Jeong Hun Lee, Won Kyong Cho, Sang Hyun Moh

**Affiliations:** 1 Plant Cell Research Institute of BIO-FD&C Co., Ltd., Incheon 21990, Republic of Korea; smchoi@biofdnc.com (S.C.); shkwon@biofdnc.com (S.H.K.); gskwon@biofdnc.com (G.S.K.); hhseo@biofdnc.com (H.H.S.); yskim@biofdnc.com (Y.S.K.); jhlee@biofdnc.com (J.H.L.); 2 Plant Health Center, Seoul National University, Seoul 08826, Republic of Korea; bioplanths@gmail.com

**Keywords:** potyvirus, HC-Pro, RNA silencing, gene expression, plant biotechnology

## Abstract

Potyviruses, a major group of plant viruses, utilize HC-Pro, a multifunctional protein, to suppress RNA silencing, a crucial plant defense mechanism. While HC-Pro’s role in RNA silencing suppression has been studied in several potyviruses, the specific mechanisms and interactions of HC-Pro from bean yellow mosaic virus (BYMV), a potyvirus with a broad host range, remain poorly understood. To address this knowledge gap, this study aimed to investigate the role of P1 and HC-Pro from BYMV in enhancing gene expression and suppressing RNA silencing in *Nicotiana benthamiana*. The findings revealed that BYMV HC-Pro significantly enhanced reporter transgene expression, likely through the suppression of RNA silencing pathways. This effect was further amplified by the presence of the P1 protein, another viral component. Analysis of HC-Pro mutants revealed that the conserved FRNK box within HC-Pro is crucial for its suppression activity and its ability to enhance gene expression. Furthermore, HC-Pro significantly downregulated the expression of key RNA silencing-related genes, including *DCL2*, *DCL4*, *RDR6*, *AGO1-1*, *AGO1-2*, and *AGO2*. These findings demonstrate that the BYMV P1::HC-Pro complex serves as a potent suppressor of RNA silencing and a promising tool for enhancing gene expression in plants. The results have significant implications for developing novel strategies in plant biotechnology, particularly for the production of high-value recombinant proteins.

## 1. Introduction

Potyviruses constitute a significant group of plant viruses that cause substantial economic losses globally, adversely affecting crop yields and quality [1,2]. These viruses possess a complex genome, which encodes several proteins, including the highly conserved multifunctional protein HC-Pro [3]. HC-Pro plays a crucial role in viral replication, movement, and pathogenicity [3]. One of its primary functions is the suppression of RNA silencing, an essential plant defense mechanism that targets viral RNAs for degradation [4]. RNA silencing involves the recognition and cleavage of double-stranded RNA by Dicer-like proteins (DCLs) into viral small interfering RNAs (vsiRNAs) [5]. These vsiRNAs are subsequently incorporated into the RNA-induced silencing complex (RISC), which guides the degradation or translational repression of complementary mRNAs [6].

To counteract RNA silencing, potyviruses have evolved sophisticated strategies, with HC-Pro acting as a key suppressor. Previous studies on various potyviruses have demonstrated that HC-Pro interacts with components of the RNA silencing machinery, including DCLs, Argonaute proteins (AGOs), and RNA-dependent RNA polymerases (RDRs) to inhibit RNA silencing [7]. HC-Pro-mediated silencing suppression can occur through multiple mechanisms, including protein–protein interactions, direct RNA binding, and interference with siRNA biogenesis or function. The efficacy of HC-Pro as an RNA silencing suppressor varies among different potyviruses, with distinct clades within the potyvirus genus suggesting potential variations in HC-Pro function and virulence [8]. Although these general mechanisms are well-established, the specific role and potential biotechnological applications of HC-Pro from bean yellow mosaic virus (BYMV), a potyvirus with a broad host range, remain largely unexplored [9]. Therefore, investigating the potential of BYMV P1 and HC-Pro in enhancing recombinant protein expression in plants could provide valuable insights for advancing plant biotechnology applications.

Genetic transformation, a cornerstone of modern biotechnology, involves introducing foreign genes into living cells, enabling the production of valuable proteins in various organisms [10]. This technology, first demonstrated by the successful expression of a bacterial gene in tobacco in 1984, has revolutionized fields such as agriculture and medicine [11]. Plant-based platforms for producing high-value proteins, including therapeutics and industrial enzymes, are gaining increasing attention due to their cost-effectiveness compared to animal cell culture and microbial fermentation systems [12]. However, achieving stable and high-level expression of introduced genes in plants presents several challenges. One significant obstacle is the plant’s innate defense mechanisms, particularly RNA silencing, which can suppress the expression of foreign genes. To overcome this barrier, several studies have investigated the use of viral suppressors of RNA silencing, such as HC-Pro from potyviruses [13,14]. HC-Pro has been shown to interfere with various components of the RNA silencing machinery, facilitating the efficient expression of viral genes. However, many potyviruses exhibit narrow host ranges, limiting the applicability of their HC-Pro proteins across diverse plant systems [15].

BYMV, a member of the *Potyviridae* family, is a potyvirus known for its exceptionally broad host range, infecting nearly 200 species across 14 plant families [15,16]. This extensive host range suggests that BYMV has evolved unique strategies to overcome host defenses, making it a compelling subject for studying viral–host interactions and developing novel biotechnological tools. Like other potyviruses, BYMV encodes the highly conserved HC-Pro protein, which plays a crucial role in RNA silencing suppression. These characteristics make BYMV HC-Pro a promising candidate for the development of a silencing suppression vector.

In this study, we investigated the role of BYMV P1 and HC-Pro in enhancing gene expression and suppressing RNA silencing in *Nicotiana benthamiana*. To understand the evolutionary relationships of BYMV, we conducted a phylogenetic analysis of 98 potyviruses using HC-Pro protein sequences and characterized the conserved FRNK box within the HC-Pro protein sequence. Additionally, we assessed the impact of BYMV HC-Pro on transgene expression using transient expression assays with a GFP reporter gene in *N. benthamiana* leaves. Our study also examined the roles of the P1 protein and the FRNK box in modulating gene expression and RNA silencing suppression by analyzing various HC-Pro constructs. These analyses provided insights into the mechanisms by which BYMV HC-Pro enhances gene expression and suppresses RNA silencing. The findings have significant implications for understanding potyvirus pathogenesis and developing novel strategies for controlling economically important plant pathogens. Furthermore, our findings may contribute to the development of improved plant expression systems that leverage the RNA silencing suppression activity of BYMV P1::HC-Pro to achieve stable and high-level expression of target proteins across diverse plant hosts.

## 2. Materials and Methods

### 2.1. Phylogenetic Analysis and Conservation Assessment of Potyvirus HC-Pro Protein Sequences

A comprehensive search for complete genomes of potyviruses was conducted using the National Center for Biotechnology Information (NCBI) GenBank reference genome sequence database (https://www.ncbi.nlm.nih.gov/refseq/ accessed on 13 December 2022). The search yielded 98 potyvirus genomes. Sequence alignment was performed using MAFFT version 7.526 with the auto option, enabling the extraction of the HC-Pro protein sequences from the aligned data [17]. To identify the best-fit substitution model for the aligned HC-Pro sequences, we utilized IQ-Tree version 2.3.6, employing ModelFinder and the Bayesian information criterion (BIC) [18]. This analysis determined that the optimal model for the HC-Pro protein sequences was LG+I+G4. Subsequently, a phylogenetic tree was constructed using IQ-TREE with the LG+I+G4 model to infer evolutionary relationships among the potyviruses. The reliability of the resulting tree topology was assessed through 1000 bootstrap replicates. The phylogenetic tree was visualized using FigTree version 1.4.4, providing a clear representation of the evolutionary relationships among the viruses.

In addition to the phylogenetic analysis, we conducted a conservation analysis of 25 HC-Pro amino acid sequences within group A. For this purpose, WebLogo 3 was employed to generate a sequence logo representation of the aligned sequences, effectively highlighting highly conserved amino acid positions [19].

### 2.2. Isolation of BYMV P1 and HC-Pro Genes

To develop a novel gene silencing suppressor, we isolated the P1 and HC-Pro gene regions from BYMV-infected gladiolus through a molecular cloning approach. The target region, spanning from the 5′-UTR to the beginning of the P3 gene, was amplified using PCR and subsequently TA-cloned into a pGEM T-Easy vector. Following transformation into Escherichia coli TOP10F’ strain, sequence confirmation was performed. Primers were carefully designed based on the HC-Pro RNA sequence from NCBI GenBank (accession number: AM884180.1), with the forward primer sequence 5′-CGCATTCAGACCTTCAAACA-3′ and the reverse primer sequence 5′-CGAATGGCTCGTGCTCTATTATCCT-3′. PCR amplification was conducted using Phusion High-Fidelity DNA Polymerase under optimized thermal cycling conditions. The protocol included an initial denaturation step at 98 °C for 30 s, followed by 30 cycles of denaturation at 98 °C for 10 s, annealing at 50 °C for 10 s, and extension at 72 °C for 1 min. The reaction concluded with a final extension step at 72 °C for 1 min, with samples preserved at 4 °C. This systematic approach ensured the precise isolation and amplification of the BYMV P1 and HC-Pro gene regions, laying the foundation for advanced molecular research and the development of gene silencing suppressors.

### 2.3. Construction of BYMV P1 and HC-Pro Gene Vector

Two plant transformation vectors were developed based on the pCAMBIA backbone for Agrobacterium-mediated transformation. The first vector, PlantGEM-V9-10, was constructed by inserting a codon-optimized human epithelial growth factor (EGF) gene fused with enhanced green fluorescent protein (eGFP) downstream of the pCaMV 35S promoter. To create the second vector, PlantGEM-V9-1A, we modified PlantGEM-V9-10 by inserting the BYMV P1 and HC-Pro gene region in parallel to the target gene expression cassette. This modification was achieved by replacing the antibiotic resistance gene between the dual CaMV35S promoter(enhancer) and CaMV polyA using the *Xho*I restriction enzyme (NEB). In our experiments, PlantGEM-V9-10 served as the control vector without HC-Pro, while PlantGEM-V9-1A, containing P1 and HC-Pro, was used for transient gene expression in plants through Agrobacterium-mediated transformation.

### 2.4. Construction of BYMV P1 and HC-Pro Vectors with FRNK Motif Modifications

To investigate the role of the RNA silencing suppressor HC-Pro, we developed several vectors by modifying the FRNK motif in its central domain and examining changes in protein expression with and without the *P1* gene. The V9-10 vector was generated through *Xho*I digestion and self-ligation of the V9-1A vector. The V9-1B vector was created by digesting the V9-1A vector with *Xho*I and *Cla*I, followed by the insertion of only the HC-Pro gene (excluding *P1*) using Q5 DNA polymerase. Additionally, site-directed mutagenesis of a key residue in the FRNK motif was performed through PCR using specific primers (Table S1). We generated V9-1A delta and V9-1B delta vectors, in which the arginine (R) in the FRNK motif was substituted with isoleucine (I). The V9-1A delta vector was created by digesting the V9-1A vector with *Xho*I, followed by PCR-based insertion using specific primers (V9-1A_IF_For and ByHcPR-delta_IF_Rev, ByHcPR-delta_IF_For and V9-1A_IF_Rev). The V9-1B delta vector was constructed using the same method, with the V9-1B_IF_For primer substituted in the PCR process. These modifications facilitated the investigation of how alterations in HC-Pro influence protein expression, providing insights into its role as an RNA silencing suppressor. The primers used for various PCR combinations are summarized in Table S2.

### 2.5. Agroinfiltration Procedure for N. benthamiana Plants

All *N. benthamiana* plants were cultivated under a 16 h light/8 h dark photoperiod, using a light-emitting diode (185–200 μmol m^−2^ s^−1^) at 25 °C. Four-week-old plants were selected for agroinfiltration. The plasmids were transformed into *A. tumefaciens* strain GV3101 and utilized for syringe agroinfiltration. The *A. tumefaciens* strain GV3101 was cultured in a lysogeny broth (LB) medium supplemented with kanamycin (50 mg/L), gentamycin (50 mg/L), and rifampicin (25 mg/L) at 200 rpm and 28 °C. Once the culture reached an optical density at 600 nm (OD_600_) of 0.8, it was diluted 50-fold in fresh LB medium containing 20 µM acetosyringone and cultured at 200 rpm and 28 °C until the OD_600_ reached 0.6. *Agrobacterium* cells were harvested by centrifugation at 3500× *g*, resuspended in infiltration buffer (5 mM MES pH 5.6, 5 mM MgCl_2_, 200 μM acetosyringone, pH = 5.6), adjusted to an OD_600_ of 0.2, and used for syringe agroinfiltration. The suspension (200 μL) was infiltrated into *N. benthamiana* leaves using a needleless syringe. Leaf samples were collected at 24, 48, and 72 h post-infiltration for further analysis. Successful infiltration was confirmed by observing the spread of the wetting area on the leaf surface.

### 2.6. Protein Extraction and Western Blot Analysis for Agroinfiltrated N. benthamiana Samples

Protein extraction was performed by lysing the infiltrated leaf samples in SDS lysis buffer containing 10 mM EDTA (pH 8.0), 10 mM β-Mercaptoethanol, 4 mM DTT, 0.1% Triton X-100, 0.1% SDS, 250 mM sucrose, and 10% glycerol. Phosphatase inhibitor cocktails (Millipore Sigma, Burlington, MA, USA) were added to the lysis buffer to prevent degradation. Protein concentrations were determined using the Bradford protein assay from Bio-Rad (Hercules, CA, USA). Subsequently, 20 μg of the lysates were loaded onto 12% acrylamide gels and transferred to PVDF membranes (Thermo Fisher Scientific, Waltham, MA, USA) using the tank blotting method (Bio-Rad). Ponceau S staining solution (Thermo Fisher Scientific) was used to stain the membranes to normalize protein transfer efficiency. Membranes were blocked in 5% milk dissolved in TBS containing 0.1% Tween 20 and incubated overnight at 4 °C with primary antibodies: anti-EGF (ab206423, 1:1000) from Abcam (Cambridge, MA, USA), anti-GFP monoclonal antibody (MA5-15256, 1:5000) from Invitrogen (Carlsbad, CA, USA), and anti-actin (AS21 4615-10, 1:5000) from Agrisera (Vannas, Sweden). Following primary antibody incubation, HRP-conjugated anti-rabbit or anti-mouse secondary antibodies (Vector Laboratories, Burlingame, CA, USA) were applied. The membranes were developed using Supersignal West Femto chemiluminescence reagents (Thermo Fisher Scientific, 34577), and signals were visualized using a ChemiDoc XRS+ chemiluminescence scanner (Bio-Rad).

### 2.7. RNA Extract and Quantitative Real-Time RT-PCR

Total RNA was isolated from plant tissues using the RNeasy Plant Mini Kit (QIAGEN, Hilden, Germany, Cat. No. 74904), following the manufacturer’s instructions. Reverse transcription was performed using the SuperPrep™ Cell Lysis and RT Kit (TOYOBO, Osaka, Japan, Code No. SCQ-101S) with 2 μg of total RNA to synthesize first-strand cDNA.

RT-qPCR analysis was conducted using a Rotor-Gene Q real-time PCR System (QIAGEN, Hilden, Germany) in 72-well blocks. The reactions were performed using THUNDERBIRD™ SYBR^®^ qPCR Mix Kit (TOYOBO, Osaka, Japan, Code No. QPS-201), in a total volume of 20 μL. Each reaction was performed in triplicate, with at least two biological replicates included. Absolute quantification was performed using standard curves generated from a diluted series of cDNA containing individual genes. Transcript levels of each gene were normalized to the internal control *NbPP2A* or *NbGAPDH* using the 2^−ΔΔCT^ method.

### 2.8. Quantification of Human EGF Using ELISA

Total soluble protein samples were diluted 1:20 prior to each assay and evaluated using the Human EGF ELISA kit (Quantikine ELISA, DEG00) from R&D Systems (Minneapolis, MN, USA), following the manufacturer’s protocol. Each well was loaded with 10 μg of total protein in a 200 μL volume, resulting in a final concentration of 50 ng/μL. The assay’s detectable range for EGF levels was 3.9–250 pg/mL. Absorbance was measured at 450 nm using the Multiskan^®^ FC 1 Front microplate spectrophotometer (Thermo Fisher Scientific, Waltham, MA, USA).

## 3. Results

### 3.1. Phylogenetic Analysis and Characterization of BYMV HC-Pro in Relation to Other Potyviruses

We collected all available BYMV complete genome sequences to conduct a comprehensive phylogenetic analysis of BYMV in relation to other potyviruses, with a particular focus on HC-Pro protein sequences. According to the latest taxonomy release by the International Committee on Taxonomy of Viruses (ICTV), there are currently 212 species in the genus *Potyvirus*. Based on the HC-Pro protein sequences of 98 potyviruses, the phylogenetic analysis classified these viruses into five groups (Figure S1). BYMV was closely related to clover yellow vein virus in group A, which comprised 25 potyviruses. Group B, containing 9 potyviruses, was the smallest group, whereas group D, with 29 potyviruses, was the largest group. Groups D and E, wherein HC-Pro proteins were used for silencing vector construction, including zucchini yellow mosaic virus and tobacco etch virus.

**Figure 1:**
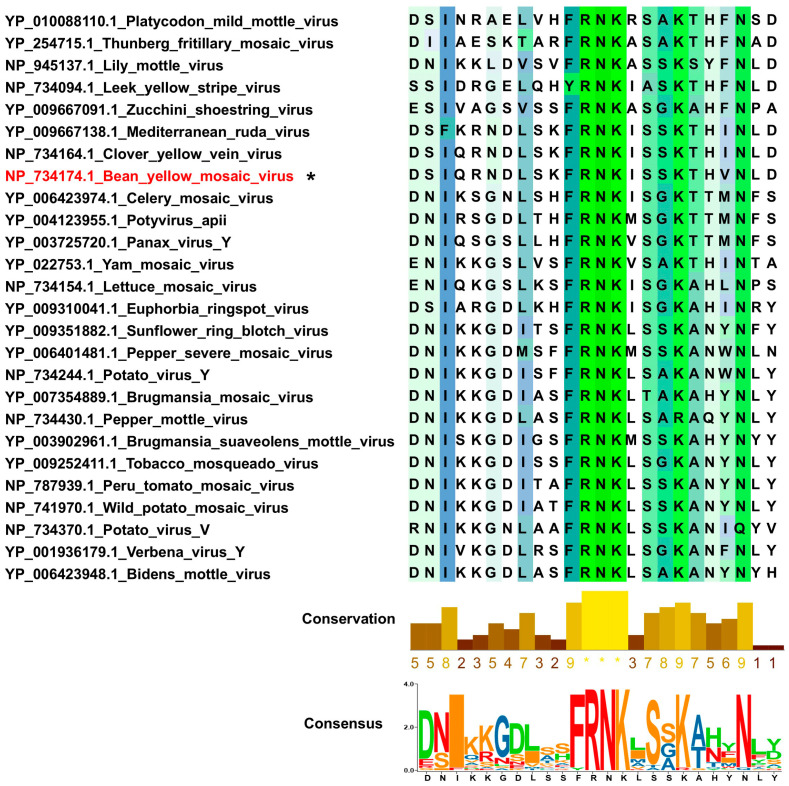
Alignment of HC-Pro protein sequences from 25 potyviruses in group A. Highly conserved amino acid sequences are represented using the WebLogo 3 program. The bean yellow mosaic virus strain used in this study is indicated by an asterisk (*).

To identify and characterize the conserved FRNK box within HC-Pro, we aligned the HC-Pro protein sequences of the 25 potyviruses in group A. This analysis revealed that most potyviruses contain the highly conserved FRNK box, which is associated with symptom severity and RNA silencing suppression, except the leek yellow stripe virus, which contains the YRNK motif instead (Figure 1). Comparing BYMV’s HC-Pro sequence with those of other potyviruses in the same phylogenetic group provided valuable insights into its evolutionary relationships and functional similarities. This analysis also assessed the potential of BYMV HC-Pro as a candidate for developing a silencing suppression vector, considering its close phylogenetic relationship with other potyviruses whose HC-Pro proteins have been successfully used for this purpose.

### 3.2. BYMV HC-Pro Enhances GFP Expression in N. benthamiana Leaves

**Figure 2:**
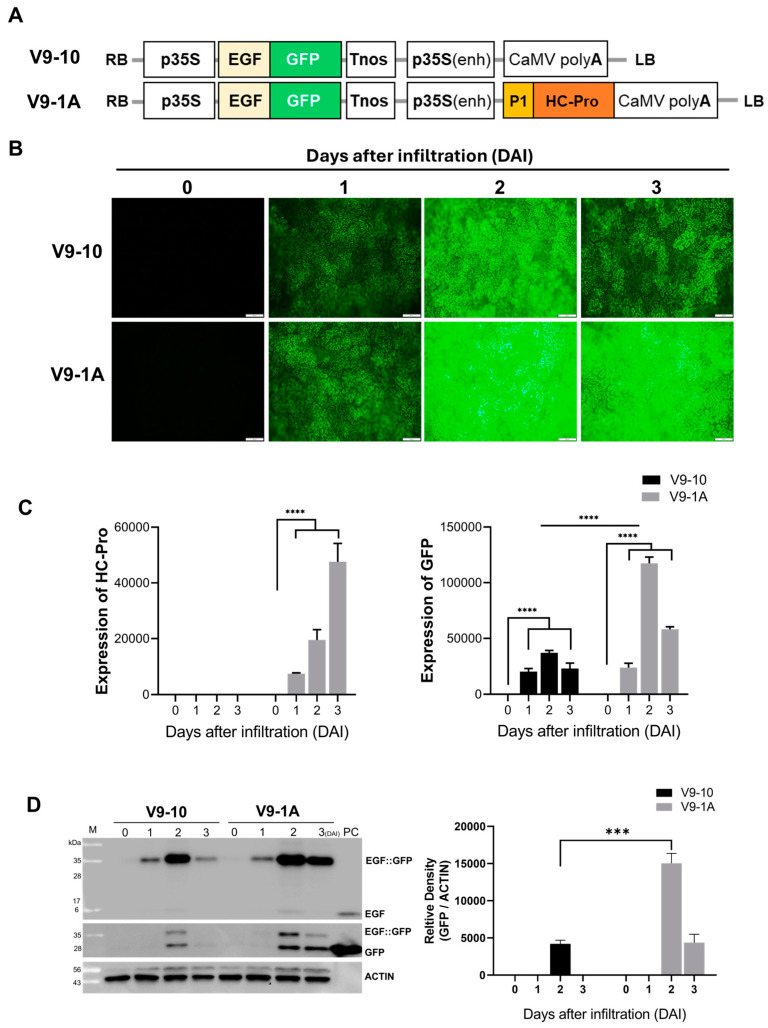
BYMV P1 and HC-Pro enhance GFP expression in *N. benthamiana* leaves. (**A**) Schematic representation of constructs used for transient expression. (**B**) GFP fluorescence in leaves infiltrated with V9-10 (without P1::HC-Pro) and V9-1A (with P1::HC-Pro) at different time points post-infiltration. (**C**) mRNA expression levels in leaves at 0, 1, 2, and 3 days after infiltration (DAI) with V9-10 and V9-1A. GFP and HC-Pro gene expression was analyzed using RT-qPCR, with serine/threonine protein phosphatase 2A (NbPP2A) included as an internal control. PC indicates recombinant EGF and GFP protein. Data are presented as means ± SD from three biological replicates. (**D**) Western blot analysis of EGF and GFP expression, with ImageJ-(Version 1.54j) based measurement quantification showing GFP expression at 1, 2, and 3 DAI. ACTIN served as a loading control. Asterisks indicate statistical significance (*** *p* < 0.001, **** *p* < 0.0001). The asterisk above the black line indicates significance between the samples.

To investigate the role of BYMV HC-Pro in enhancing gene expression, we generated two constructs for transient expression in *N. benthamiana* leaves (Figure 2A): V9-10 expressing EGF::GFP (epidermal growth factor and green fluorescent protein fusion) alone, and V9-1A, expressing both EGP::GFP and BYMV P1::HC-Pro. GFP fluorescence was observed in leaves infiltrated with both constructs, confirming successful transgene expression (Figure 2B). In leaves infiltrated with the control (V9-10), weak GFP fluorescence was detected at 1 day after infiltration (DAI), with fluorescence intensity increasing by 2 DAI, followed by a decline at 3 DAI. In contrast, leaves infiltrated with V9-1A exhibited a slight increase in GFP fluorescence compared to the control at 1 DAI, a significant increase at 2 DAI, which was maximized during the observation period, and a slight decrease at 3 DAI. The fluorescence signal was significantly higher in V9-1A infiltrated leaves compared to that in the control leaves throughout the observation period. Leaves infiltrated with V9-1A exhibited significantly higher GFP fluorescence compared to those with V9-10 at 2 and 3 DAI, suggesting that P1 and HC-Pro enhance GFP expression, implying that GFP expression was strongly induced in response to HC-Pro while maintaining the elevated expression level afterward.

To evaluate whether the presence of P1 and HC-Pro enhances EGF::GFP expression compared to the control without P1::HC-Pro, qRT-PCR analysis was conducted. The results revealed that P1 and HC-Pro significantly increased GFP mRNA levels in leaves infiltrated with V9-1A as DAI progressed; however, no HC-Pro expression was detected in V9-10 (Table S3 and Figure 2C). The quantification of GFP mRNA levels through qRT-PCR analysis revealed a significant increase in GFP mRNA accumulation in leaves infiltrated with V9-1A compared to V9-10 (Figure 2C). Specifically, without P1::HC-Pro, GFP expression was high at 2 DAI. With P1 and HC-Pro, GFP expression was also the highest at 2 DAI. Notably, leaves infiltrated with V9-1A exhibited higher levels of GFP mRNA compared to those with V9-10, consistent with the fluorescence observations. GFP expression in the presence of P1::HC-Pro peaked at 2 DAI.

Following this, Western blot analysis of EGF protein levels in the same samples was performed using specific antibodies (Figure 2D). Consistent with the qRT-PCR results, Western blot analysis revealed significantly higher levels of GFP protein in leaves infiltrated with V9-1A compared to V9-10 (Figure 2D). Notably, at 2 DAI, the expression level of GFP in the vector containing P1::HC-Pro was more than 3.5-fold higher, with statistical significance (*p* < 0.001). Immunoblot analyses demonstrated that EGF protein accumulation was significantly elevated at 2 DAI and remained elevated at 3 DAI in the presence of the co-expressed P1::HC-Pro gene.

### 3.3. BYMV HC-Pro Suppresses Post-Transcriptional Gene Silencing (PTGS) in N. benthamiana

**Figure 3:**
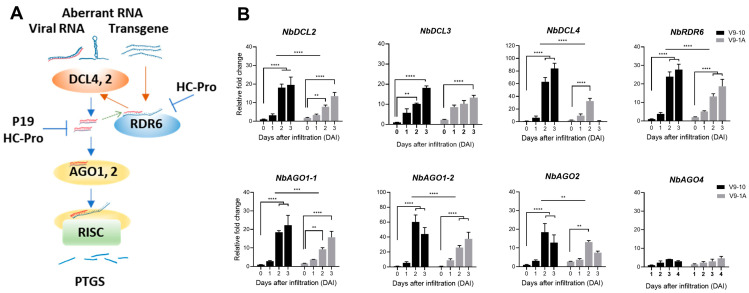
PTGS pathway and gene expression changes induced by BYMV HC-Pro protein. (**A**) Schematic representation of the PTGS pathway. Aberrant RNA (viral or transgene) is recognized by Dicer-like proteins (DCLs), which cleave the RNA into small interfering RNAs (siRNAs). siRNAs are loaded into Argonaute (AGO) proteins to form the RNA-induced silencing complex (RISC), which targets the mRNA for degradation or translational inhibition. HC-Pro and P19 proteins suppress PTGS by targeting different components of the pathway. (**B**) Relative expression levels of PTGS-related genes in *N. benthamiana* leaves infiltrated with BYMV P1 and HC-Pro at different time points post-infiltration. Data are presented as mean ± SD (*n* = 3). Statistical significance was determined using Student’s *t*-test (** *p* < 0.01, *** *p* < 0.001, **** *p* < 0.0001).

Post-transcriptional gene silencing (PTGS) is a vital plant defense mechanism that targets aberrant RNAs, such as viral RNAs or transgenes (Figure 3A). This process involves Dicer-like proteins (DCLs), which cleave double-stranded RNA into small interfering RNAs (siRNAs). These siRNAs are subsequently loaded into Argonaute (AGO) proteins, forming the RNA-induced silencing complex (RISC). RISC then targets complementary mRNAs for degradation or translational inhibition.

To investigate the impact of the viral suppressor of RNA silencing, HC-Pro, on PTGS components, we assessed the expression levels of key PTGS genes in *N. benthamiana* leaves infiltrated with P1::HC-Pro (Table S3 and Figure 3B). Quantitative RT-PCR analysis revealed that HC-Pro significantly downregulated the expression of *DCL2* and *DCL4* at 2 and 3 DAI. Additionally, P1::HC-Pro significantly reduced the expression of *DCL3*, *RDR6*, *AGO1-1*, *AGO1-2*, and *AGO2* at various time points. No significant effect was observed on *AGO4* expression, which functions in the transcriptional gene silencing (TGS) pathway. These findings suggest that P1::HC-Pro suppresses PTGS by targeting multiple components of the siRNA biogenesis and effector pathways, including DCLs, RDR6, and AGOs.

### 3.4. Characterization of P1-Mediated Translational Enhancement of HC-Pro

**Figure 4:**
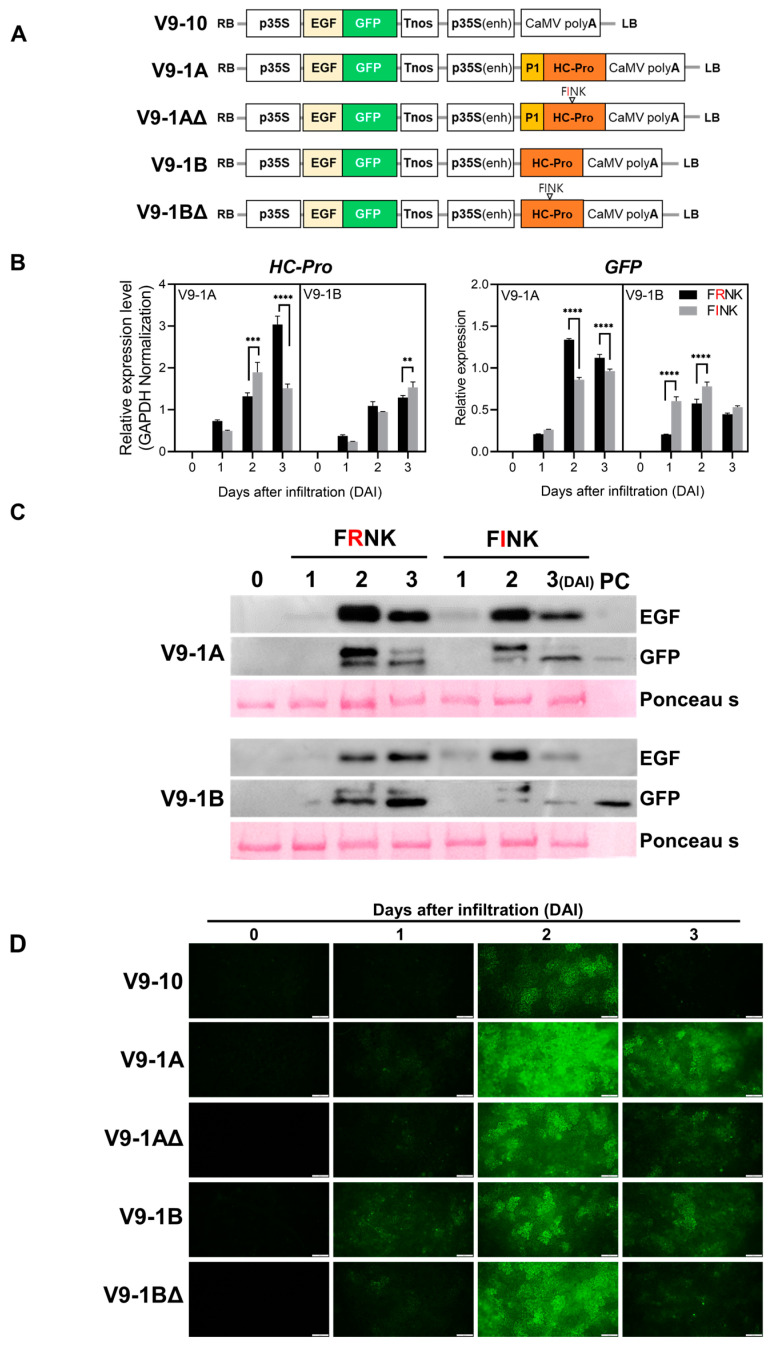
Analysis of P1-mediated HC-Pro expression and its effect on protein accumulation. (**A**) Schematic representation of the constructs used in this study. V9-10 is the control vector. V9-1A contains the P1 and HC-Pro protein expression cassette. V9-1AΔ(delta) features the substitution of FRNK with FINK. V9-1B expresses only HC-Pro without the P1 protein. V9-1BA is identical to V9-1A but lacks the P1 protein. (**B**) Quantitative RT-PCR analysis of HC-Pro mRNA levels in *N. benthamiana* leaves infiltrated with *A. tumefaciens* GV3101 carrying the indicated constructs. Data are expressed as relative fold change compared to the control (V9-10). Error bars represent standard deviations from three independent experiments. Statistical significance was determined using Student’s *t*-test (** *p* < 0.01, *** *p* < 0.001, **** *p* < 0.0001). (**C**) Western blot analysis of EGF and GFP protein levels in *N. benthamiana* leaves infiltrated with A. tumefaciens GV3101 carrying the indicated constructs. EGF and GFP protein levels were detected using specific antibodies. (**D**) Confocal microscopy images of *N. benthamiana* leaves infiltrated with A. tumefaciens GV3101 carrying the indicated constructs. GFP fluorescence was visualized using a fluorescence microscope.

To further investigate the role of P1 in translational enhancement, we generated three additional constructs (Figure 4A). The V9-1AΔ construct contained the FRNK substitution in place of the FINK motif in the HC-Pro protein. The V9-1B construct was generated by deleting the P1 gene from the V9-1A construct. The V9-1BΔ construct was identical to V9-1AΔ but lacked the P1 gene. qRT-PCR was performed to determine changes in HC-Pro and GFP expression in the presence or absence of the P1 gene and the FINK mutation (HC-Pro-ΔFINK) (Figure 4B). In wild-type (WT) samples with functional HC-Pro, HC-Pro gene expression was detectable and increased over time (1 to 3 DAI). The presence of the P1 gene promoted HC-Pro gene expression as the infiltration period progressed.

In HC-Pro-ΔFINK samples, HC-Pro gene expression was significantly increased in constructs containing the FINK mutation (Table S4 and Figure 4B). At 3 DAI, HC-Pro gene expression was significantly higher in the FINK mutant compared to the control containing FRNK (*p* < 0.01). In contrast, HC-Pro gene expression peaked at 2 DAI in constructs containing both P1 and HC-Pro genes. HC-Pro gene expression in the FINK mutant decreased at 3 DAI compared to 2 DAI. In constructs containing P1 and HC-Pro genes, HC-Pro gene expression was significantly higher in the FINK mutant than in the FRNK control at 2 DAI (*p* < 0.001), but lower at 3 DAI (*p* < 0.0001).

In constructs expressing only HC-Pro, GFP expression peaked at 2 DAI for both FRNK and FINK constructs, with FINK exhibiting significantly higher expression at 1 and 2 DAI. In constructs containing P1 and HC-Pro genes, GFP expression significantly increased at 2 and 3 DAI compared to 1 DAI. GFP expression in the FRNK domain construct was significantly higher than in the FINK domain construct at 2 and 3 DAI (*p* < 0.0001). Across all conditions, GFP expressions consistently peaked at 2 DAI, regardless of the FRNK or FINK domain.

Western blot analysis was subsequently performed to assess changes in EGF and GFP protein expression caused by the P1 gene and the FINK mutation (Figure 4C). In the V9-1A construct containing both P1 and HC-Pro genes, EGF protein expression peaked at 2 DAI for both FRNK and FINK mutants among the three time points. GFP expression followed a similar pattern, exhibiting strong expression at 2 DAI. Compared to the positive control, EGF protein expression was comparable at 1 DAI. However, GFP expression at 1 DAI was not detected in either the FRNK or FINK constructs. In the V9-1B construct containing only HC-Pro, EGF protein expression increased over time in the WT construct containing the FRNK domain. Conversely, EGF protein expression peaked at 2 DAI in the construct containing the FINK domain. GFP expression also increased over time in the WT containing the FRNK domain. However, GFP expression was significantly lower in constructs containing the FINK domain compared to the positive control.

Fluorescence microscopy was performed to examine GFP fluorescence in *N. benthamiana* leaves infiltrated with different constructs (Figure 4D). At 1 DAI, only the V9-1A construct containing both P1 and HC-Pro genes exhibited visible GFP expression, whereas no detectable GFP signal was observed in other constructs. At 2 DAI, most constructs displayed strong GFP fluorescence. Among the five constructs, V9-1B containing P1 and HC-Pro genes exhibited the strongest GFP fluorescence, followed by V9-1AΔ and V9-1BΔ containing the FINK domain. At 3 DAI, V9-1B continued to exhibit the strongest GFP fluorescence, followed by V9-1B and V9-1A.

### 3.5. Differential Expression of RNA Silencing Genes in N. benthamiana Infiltrated with HC-Pro or P1::HC-Pro Constructs Containing FRNK or FINK Domains

**Figure 5:**
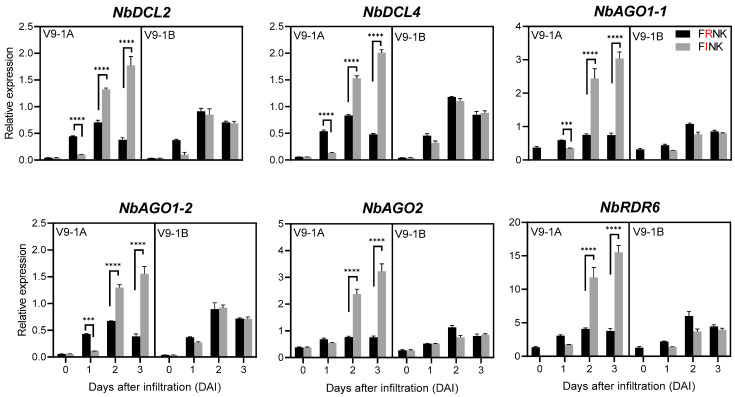
Expression of RNA silencing genes in *N. benthamiana* infiltrated with HC-Pro or P1::HC-Pro constructs containing FRNK or FINK domains. Relative expression levels of DCL2, DCL4, AGO1-1, AGO1-2, AGO2, and RDR6 in *N. benthamiana* leaves infiltrated with constructs containing HC-Pro or P1 and HC-Pro, with either FRNK or FINK domains, at 0, 1, 2, and 3 days after infiltration (DAI). Error bars represent standard deviation (*n* = 3). Statistical significance compared to FRNK and FINK: *** *p* < 0.001, **** *p* < 0.0001.

The expression of six RNA silencing genes (DCL2, DCL4, AGO1-1, AGO1-2, AGO2, and RDR6) was analyzed in *N. benthamiana* leaves infiltrated with different constructs (Figure 5). In plants expressing HC-Pro alone, the highest expression levels of all six genes were observed at 2 DAI, followed by 3 DAI and 1 DAI. No significant differences in gene expression were detected between constructs containing FRNK or FINK domains.

In contrast, plants expressing both P1 and HC-Pro genes exhibited dynamic changes in gene expression. In FRNK-containing constructs, DCL2, DCL4, and AGO1-2 showed peak expression at 2 DAI, while AGO1-1, AGO2, and RDR6 displayed similar expression levels at 2 and 3 DAI (Table S4 and Figure 5). In FINK-containing constructs, the expression of all six genes progressively increased over time. At 2 and 3 DAI, the expression levels in FINK-containing constructs were significantly higher than those in FRNK-containing constructs for all genes (*p* < 0.0001). However, except for RDR6, the expression levels of the remaining five genes were significantly higher in FRNK-containing constructs compared to FINK-containing constructs at these time points (*p* < 0.001).

**Figure 6:**
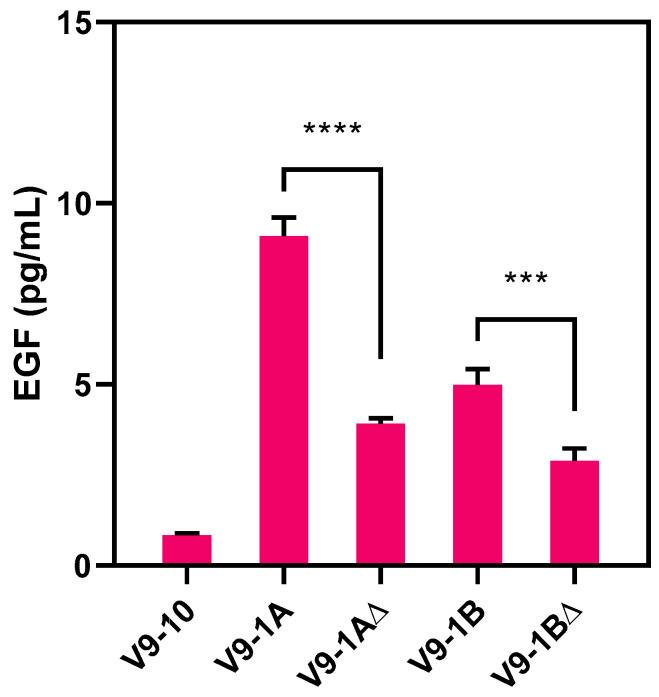
Comparison of EGF accumulation facilitated by HC-Pro and P1::HC-Pro in combination with FRNK mutation. EGF content was measured using the human EGF ELISA kit in tobacco leaves infiltrated with A. tumefaciens GV3101 carrying EGF::GFP with or without BYMV_P1. Error bars represent standard deviation (*n* = 3). Statistical significance compared to FRNK and FINK: *** *p* < 0.001, **** *p* < 0.0001. Asterisks located above the black line indicate significance between the 2 DAI samples.

Quantification of EGF protein levels revealed a significant reduction in EGF production in leaves infiltrated with V9-1A and V9-1B constructs compared to those containing FINK mutations (Figure 6). Furthermore, a decrease in EGF expression was observed in constructs with FINK mutations, confirming that the FRNK region is critical for the functional role of the protein. Co-expression of V9-1A resulted in the highest accumulation of EGF protein at 2 DAI, reaching 9.09 pg/mL, an approximately 10-fold increase compared to V9-10 (0.831 pg/mL). The essential role of the PI region within the HC-Pro protein was further demonstrated by a 1.8-fold reduction in EGF protein expression compared to V9-1A and V9-1B constructs. These findings highlight that the co-expression of the BYMV P1::HC-Pro (V9-1A) gene suppresses target gene silencing and significantly enhances the synthesis of the target protein product.

## 4. Discussion

Plants utilize an RNA-based adaptive antiviral immunity mechanism wherein small RNAs derived from both viral RNA strands guide an AGO nuclease [20]. Nearly all plant viruses produce multifunctional proteins that act as suppressors of RNA silencing [21]. Several plant RNA viruses encode proteins that inhibit RNA silencing, a critical plant defense mechanism. Examples include P1/HC-Pro of tobacco etch potyvirus (TEV), 2b of cucumber mosaic virus (CMV), p25 of potato virus X (PVX), and the coat protein (CP) of citrus tristeza virus (CTV) [22]. Additionally, geminiviruses and their associated DNA satellites also encode viral suppressors of RNA silencing [23].

Our study focused on BYMV as a source of viral suppressors, specifically P1 and HC-Pro, for several key reasons. First, BYMV exhibits a broad host range, making its silencing suppressors potentially applicable across diverse plant species for practical applications. Additionally, developing expression vectors based on novel viruses is crucial for commercial applications, and BYMV provides an opportunity to harness previously unutilized viral components. Although P1 and HC-Pro from other potyviruses have been extensively studied, investigating these proteins in the context of BYMV broadens our understanding of their functions across different viral species. Ultimately, our research aims to expand the repertoire of viral suppressors for gene silencing applications while advancing our knowledge of potyvirus biology.

A comprehensive phylogenetic analysis of 98 potyvirus HC-Pro protein sequences revealed five distinct groups, indicating the diversity of HC-Pro proteins within potyviruses. Notably, BYMV was found to be closely related to clover yellow vein virus (CIYVV) within group A, which comprises 25 potyviruses. CIYVV, like BYMV, can infect a wide range of plant species [24]. This finding suggests that potyviruses in group A may have a broad host range. HC-Pro is a multifunctional potyvirus protein composed of three domains: transmission (Domain I), genome amplification and RNA silencing suppression (Domain II), and movement with proteinase activity (Domain III) [25]. The FRNK box in Domain II plays a crucial role in RNA silencing suppression. Our analysis confirmed that the FRNK box is conserved across most potyviruses, except for the leek yellow stripe virus, which contains the YRNK motif. This conservation highlights the importance of the FRNK motif in RNA silencing suppression.

BYMV P1 and HC-Pro significantly enhanced GFP expression in *N. benthamiana* leaves, as evidenced by increased GFP fluorescence and mRNA levels. Quantitative RT-PCR analysis demonstrated higher GFP mRNA levels in leaves infiltrated with constructs co-expressing P1 and HC-Pro (V9-1A) compared to those expressing GFP alone (V9-10). The enhancement was most pronounced at 2 DAI, with over a two-fold increase in GFP expression compared to the control. The expression peaked at 2 DAI, likely due to the balance between the RNA silencing suppression activity of P1 and HC-Pro and the plant’s antiviral defenses. The viral proteins promote GFP mRNA stability and accumulation, with their suppressive effect most effective in the early stages of the infection [13,26]. At 1 DAI, the expression system may not be fully active, whereas at 3 DAI, plant defenses may counteract the suppressors [27]. HC-Pro binds to small RNA duplexes, including miRNAs, potentially increasing target mRNA levels during early infection [14,27]. This effect is likely strongest around 2 DAI. Additionally, Agrobacterium-mediated transient expression systems typically reach peak expression a few days post-infiltration [26]. We propose that these factors create optimal conditions at 2 DAI, where P1 and HC-Pro’s suppressor activity maximizes GFP expression before plant defenses are fully activated.

The presence of P1 significantly enhances HC-Pro expression and its subsequent protein accumulation. Quantitative RT-PCR analysis revealed that constructs containing both P1 and HC-Pro (V9-1A) exhibited significantly higher HC-Pro expression compared to those with HC-Pro alone (V9-1B). This synergistic effect was further validated by Western blot analysis, which demonstrated increased levels of EGF and GFP proteins in leaves infiltrated with V9-1A. Previous studies have documented this synergistic relationship, indicating that P1 enhances the role of HC-Pro as a pathogenicity enhancer and gene silencing suppressor. For instance, the combination of P1 and the viral 5′ non-translated region upstream of HC-Pro in a PVX vector resulted in greater RNA stability and accumulation compared to HC-Pro alone [28]. Additionally, local silencing suppression by HC-Pro was observed only when co-expressed with P1 [14]. This enhancement is likely attributed to improved translational efficiency rather than direct effects on HC-Pro stability.

The substitution of the FRNK domain with FINK in HC-Pro resulted in distinct expression patterns of the introduced transgenes. Constructs with the FINK mutation (V9-1AΔ and V9-1BΔ) exhibited a gradual accumulation of transgene products over time, likely attributable to the altered inhibition of the plant’s RNA silencing mechanism. In contrast, constructs with the intact FRNK domain (V9-1A and V9-1B) displayed peak expressions at specific time points. This difference underscores the role of HC-Pro in preserving transgene expression by suppressing RNA silencing rather than directly increasing gene expression. Notably, this effect was primarily observed in the introduced transgenes without significantly affecting the plant’s endogenous defense genes or viral genes. These differences were confirmed through quantitative RT-PCR and Western blot analyses, underscoring the critical role of the FRNK motif in HC-Pro’s function and its capacity to enhance gene expression over time. Previous studies have highlighted the significance of the FRNK motif in HC-Pro’s activity as a viral suppressor of gene silencing. For instance, the FRNK box exhibits a higher binding affinity for small RNA duplexes compared to the FINK variant, impacting symptom severity and viral accumulation [27]. Furthermore, mutations in the FRNK motif have been shown to attenuate symptoms without compromising viral infectivity, suggesting that this motif plays a pivotal role in regulating gene expression and symptom development in infected plants [29,30].

In our study, an unexpected increase in HC-Pro gene expression was observed at 3 DAI in the FINK mutant compared to the FRNK control. This unexpected finding may be attributed to a compensatory mechanism in response to the reduced functionality of the mutated HC-Pro. Despite the elevated gene expression, the FINK mutant exhibited lower efficacy in enhancing reporter gene expression compared to the WT FRNK version. This finding suggests that increased gene expression does not necessarily correlate with enhanced functional activity in RNA silencing suppression, highlighting the complex relationship between gene expression levels and protein functionality in viral suppressors of RNA silencing. Further investigation into this phenomenon could provide valuable insights into the plant’s response to altered viral proteins and the mechanisms governing RNA silencing suppression.

HC-Pro has been shown to effectively suppress post-transcriptional gene silencing (PTGS) by downregulating key components involved in siRNA biogenesis and effector pathways. qRT-PCR analysis indicated that HC-Pro significantly reduced the expression of several RNA silencing genes, including *DCL2*, *DCL4*, *RDR6*, *AGO1-1*, *AGO1-2*, and *AGO2* at various time points post-infiltration [31,32]. This broad-spectrum suppression underscored HC-Pro’s potent ability to interfere with plant defense mechanisms. The expression patterns of RNA silencing genes varied depending on the presence of P1 and the FRNK/FINK domains in HC-Pro. In plants expressing only HC-Pro, the highest expression of all examined genes was observed at 2 DAI. In plants expressing only HC-Pro, the highest expression of all examined genes was observed at 2 DAI for both FRNK- and FINK-containing constructs. In contrast, plants co-expressing P1 and HC-Pro displayed distinct patterns: FRNK-containing constructs peaked at 2 DAI, whereas FINK-containing constructs exhibited progressively increasing expression over time [13,33]. These findings highlight the complex dynamics of the plant’s response to viral suppressors of RNA silencing and the modulatory role of P1 in HC-Pro’s activity.

Although our findings highlight the potential of the BYMV P1/HC-Pro system for enhancing recombinant protein expression, it is essential to consider the potential drawbacks associated with HC-Pro’s pleiotropic effects. HC-Pro’s proteolytic activity and its interactions with host proteins could disrupt normal cellular processes in plants. Further research is required to fully elucidate the impact of these interactions on plant physiology and to develop strategies to mitigate any potential adverse effects. This may involve engineering HC-Pro variants with reduced proteolytic activity or altered host protein interactions while preserving their RNA silencing suppression function. A comprehensive risk assessment is critical before deploying this system in agricultural applications.

*Nicotiana benthamiana* was selected as the model plant for this study due to its unique genetic characteristics, particularly its defective RNA-dependent RNA polymerase 1 (RdRP1) gene. This genetic feature renders *N. benthamiana* highly susceptible to viral infections, making it an ideal model for virus-induced gene silencing (VIGS) and recombinant protein expression studies [34]. Although *N. benthamiana* is not an economically significant crop, the insights gained from this study could potentially be applied to other plant species susceptible to bean yellow mosaic virus infection. Future studies should involve testing the expression system and viral resistance mechanisms in multiple host species, including economically significant crops, to broaden the applicability of our findings.

The BYMV P1::HC-Pro system demonstrates significant potential for enhancing recombinant protein expression in plants, with implications for both crop biotechnology and virus resistance strategies. The observed synergistic interaction between P1 and HC-Pro, particularly the role of the conserved FRNK motif, suggests promising applications in improving crop yields and developing plant-based therapeutics. This system could be leveraged to overexpress beneficial traits in crops, such as drought tolerance genes, or to enhance the production of recombinant proteins for biopharmaceutical applications. Furthermore, understanding HC-Pro’s mechanism in suppressing RNA silencing pathways could contribute to the development of novel antiviral strategies, potentially conferring broad-spectrum virus resistance in crops. Future research should focus on optimizing this system for specific crop species and evaluating its efficacy under field conditions, which could significantly advance plant biotechnology applications.

In conclusion, our study demonstrates the potential of BYMV P1 and HC-Pro to enhance gene expression in plants. The synergistic interaction between these proteins, combined with the critical role of the FRNK motif in HC-Pro’s function, highlights the complex interplay of viral factors in manipulating host gene expression. These findings have significant implications for plant biotechnology. The ability of BYMV HC-Pro to enhance gene expression and suppress RNA silencing suggests its potential as a valuable tool for improving the production of recombinant proteins in plants, paving the way for the development of novel plant expression systems for high-value proteins.

## 5. Conclusions

In this study, we investigated the potential of BYMV P1 and HC-Pro proteins as enhancers of gene expression in plants. Our findings demonstrate that BYMV P1 and HC-Pro significantly enhance the expression of a reporter gene (GFP) in *N. benthamiana*. This enhancement is most pronounced at 2 DAI, likely attributable to the combined effects of P1 and HC-Pro suppressing RNA silencing during the early stages of infection, when plant defense mechanisms are still being established. We observed a synergistic interaction between P1 and HC-Pro, wherein P1 enhances the expression and subsequent protein accumulation of HC-Pro. Furthermore, the conserved FRNK motif within HC-Pro is crucial for its function as an RNA silencing suppressor and for the timing of gene expression enhancement. Our results provide valuable insights into the mechanisms underlying potyvirus-mediated gene expression enhancement. These findings suggest that BYMV P1 and HC-Pro, particularly those containing a functional FRNK motif, represent promising tools for enhancing gene expression in plants for various biotechnological applications, including improving crop yields and developing novel plant-based therapeutics.

## Data Availability

No new data were created or analyzed in this study.

## Supplementary Materials

The following supporting information can be downloaded at: https://www.mdpi.com/article/10.3390/life15030472/s1, Figure S1. The phylogenetic tree of 98 potyviruses based on HC-Pro protein sequences; Table S1. Oligonucleotide sequences for vector constructs; Table S2. Oligonucleotide sequences for quantitative real-time PCR analyses; Table S3. Raw data used for qRT-PCR for Figure 2 and Figure 3; Table S4. Raw data used for qRT-PCR for Figure 4 and Figure 5.

## Abbreviations

The following abbreviations are used in this manuscript:

BYMV	Bean yellow mosaic virus
DCL	Dicer-like protein
RdRP1	RNA-dependent RNA polymerase 1
VIGS	Virus-induced gene silencing
siRNAs	Small interfering RNAs
