# Supplementary figures and images for: Role of Bean Yellow Mosaic Virus P1 and HC-Pro in Enhancing Gene Expression and Suppressing RNA Silencing in Nicotiana benthamiana
Source: Life (Basel). 2025 Mar 15;15(3):472. doi: 10.3390/life15030472 (PMC11944257; doi:10.3390/life15030472)

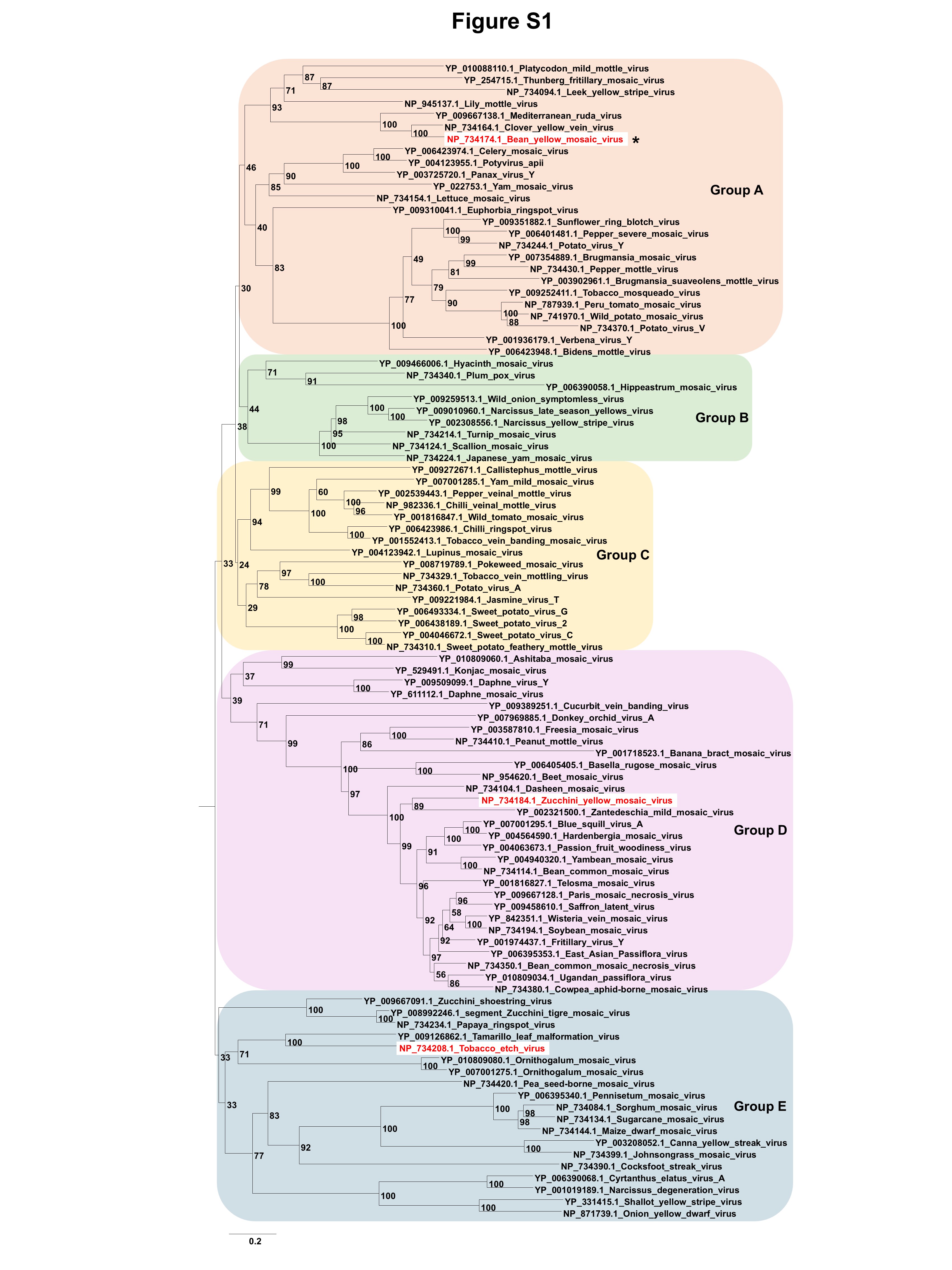

Supplement: Supplementary file 1 [file life-15-00472-s001.zip › Figure S1.jpg]
